# Predicting the Type of Tumor-Related Epilepsy in Patients With Low-Grade Gliomas: A Radiomics Study

**DOI:** 10.3389/fonc.2020.00235

**Published:** 2020-03-13

**Authors:** Yinyan Wang, Wei Wei, Zhenyu Liu, Yuchao Liang, Xing Liu, Yiming Li, Zhenchao Tang, Tao Jiang, Jie Tian

**Affiliations:** ^1^Beijing Tiantan Hospital, Capital Medical University, Beijing, China; ^2^CAS Key Laboratory of Molecular Imaging, Institute of Automation, Chinese Academy of Sciences, Beijing, China; ^3^School of Electronics and Information, Xi'an Polytechnic University, Xi'an, China; ^4^Beijing Advanced Innovation Center for Big Data-Based Precision Medicine, School of Medicine, Beihang University, Beijing, China; ^5^Engineering Research Center of Molecular and Neuro Imaging of Ministry of Education, School of Life Science and Technology, Xidian University, Xi'an, China; ^6^School of Artificial Intelligence, University of Chinese Academy of Sciences, Beijing, China; ^7^Department of Molecular Pathology, Beijing Neurosurgical Institute, Capital Medical University, Beijing, China; ^8^Center of Brain Tumor, Beijing Institute for Brain Disorders, Beijing, China; ^9^China National Clinical Research Center for Neurological Diseases, Beijing, China

**Keywords:** epilepsy type, low-grade gliomas, machine learning, radiomics, T2-weighted imaging

## Abstract

**Purpose:** The majority of patients with low-grade gliomas (LGGs) experience tumor-related epilepsy during the disease course. Our study aimed to build a radiomic prediction model for LGG-related epilepsy type based on magnetic resonance imaging (MRI) data.

**Methods:** A total of 205 cases with LGG-related epilepsy were enrolled in the retrospective study and divided into training and validation cohorts (1:1) according to their surgery time. Seven hundred thirty-four radiomic features were extracted from T2-weighted imaging, including six location features. Pearson correlation coefficient, univariate area under curve (AUC) analysis, and least absolute shrinkage and selection operator regression were adopted to select the most relevant features for the epilepsy type to build a radiomic signature. Furthermore, a novel radiomic nomogram was developed for clinical application using the radiomic signature and clinical variables from all patients.

**Results:** Four MRI-based features were selected from the 734 radiomic features, including one location feature. Good discriminative performances were achieved in both training (AUC = 0.859, 95% CI = 0.787–0.932) and validation cohorts (AUC = 0.839, 95% CI = 0.761–0.917) for the type of epilepsy. The accuracies were 80.4 and 80.6%, respectively. The radiomic nomogram also allowed for a high degree of discrimination. All models presented favorable calibration curves and decision curve analyses.

**Conclusion:** Our results suggested that the MRI-based radiomic analysis may predict the type of LGG-related epilepsy to enable individualized therapy for patients with LGG-related epilepsy.

## Introduction

World Health Organization (WHO) grade II or low-grade glioma (LGG) (1) accounts for the majority of primary brain tumors in young adults (2, 3). The majority of patients with LGG experiences tumor-related epilepsy (4, 5) that impacts their quality of life and may contribute to long-term disability (6–8). Broadly, the type of epilepsy can be generalized or focal based on its presentation (9) and require different methods of treatment. Generalized epilepsy occurs more frequently, is more severe, and requires a relatively higher dose of antiepileptic therapy with the potential for increased side effects, compared to focal epilepsy. An accurate prediction of epilepsy type that occurs in patients with brain tumors could allow customization of antiepileptic therapy.

Radiomics is a research branch in the field of medical imaging (10). Based on the rapid development of machine learning and image processing techniques, radiomic analyses have been successfully applied in the field of oncology (11–16), including glioma (17). Magnetic resonance imaging (MRI) is a routinely used diagnostic tool for glioma management. A lot of tumor information that is not recognized by human eye remains unmined (12). Radiomics can extract high-dimensional radiomic features from medical images to fully exploit the in-depth information of tumors (18). Based on T2-weighted imaging (T2WI), Liu et al. successfully predicted the occurrence of LGG-associated epilepsy by radiomic analysis (19). However, the prediction of the type of epilepsy remains to be determined.

The current study conducted a radiomic analysis to explore the relationship between quantitative radiomic features and the type of tumor-related epilepsy in patients with LGG. Precise radiomic prediction models for epilepsy type could be established and further validated using the screened features.

## Methods

### Patients

**Figure 1:**
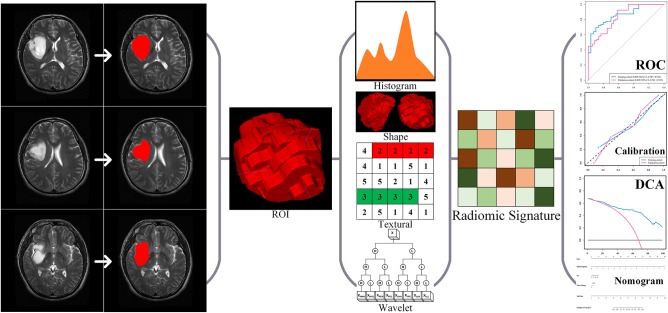
Study design and flowchart. The flowchart of the current study.

In this retrospective study, we consecutively enrolled a total of 205 patients with LGG who underwent surgery at the Beijing Tiantan Hospital from September 2012 to December 2014. The inclusion criteria of all enrolled cases were (a) pathologically confirmed grade II gliomas according to WHO criteria 2016 (20) and (b) presurgical T2-weighted imaging. The exclusion criteria of all enrolled cases were no craniotomy or stereotactic biopsy before MRI scan. The enrolled cases were allocated to either the training or the validation cohort according to the surgery time with a 1:1 ratio, i.e., the first 102 cases enrolled formed the training cohort, and the other 103 cases formed the validation cohort. Data on routine clinical variables were collected, including age, gender, tumor pathology, and epilepsy type. The present study further utilized clinical and MRI data from all enrolled cases. The ethics committee of Beijing Tiantan Hospital approved this study, and the requirement for informed consent was waived. Our study was conducted in accordance with the Declaration of Helsinki. The study design is illustrated in Figure 1.

Patients were considered to have experienced tumor-related epilepsy when a history of at least one seizure with the presence of an enduring alteration (i.e., LGG) in the brain (21) was reported. The history and type (generalized and focal) of epilepsy were evaluated by an epileptologist based on the patient's presentation according to the classification and terminology of the International League Against Epilepsy (9, 22). The epilepsy type was determined consistently for all the enrolled patients with a history of epilepsy based on the aforementioned criteria.

### Brain MRI and Tumor Segmentation

All MRI examinations were performed using a Magnetom Trio 3.0 T scanner (Siemens, Erlangen, Germany) with a 12-channel receive-only head coil scan acquisition. The T2WI parameters were as follows: repetition time (TR), 5,800 ms; echo time (TE), 110 ms; flip angle, 150°; the field of view (FOV), 240 × 188 mm^2^; voxel size, 0.6 × 0.6 × 5.0 mm^3^; and matrix, 384 × 300. The MRI data were stored in DICOM format.

Regions of interest (ROIs) of the gliomas were drawn by two neuroradiologists with more than 5 years of clinical experience with ITK-snap (www.itksnap.org). The neuroradiologists were blinded to each other's results. Gliomas were segmented on each MRI slice. We defined ROIs of the LGGs as areas of the MRI images that exhibited abnormal hyperintense signals. The intraclass correlation coefficient (ICC) was used to assess whether the segmentation results of the two doctors were significantly different. No difference was defined as ICC >0.8. In the absence of a difference, each patient would obtain a segmentation result from one of the two neuroradiologists randomly.

### Extraction of Quantitative Radiomic Features

A total of 734 radiomic features were extracted based on the research of Liu et al. (19) and Li et al. (23, 24), including 6 location features, 17 first order statistics (FOS) features, 8 shape and size features, 26 gray-level co-occurrence matrix (GLCM) features, 16 gray-level run-length matrix (GLRLM) features, 16 gray-level size zone matrix (GLSZM) features, 5 neighborhood gray-tone difference matrix (NGTDM) features, and 640 wavelet features.

The location features were extracted based on our previous research (19) using (a) polar coordinates parameters (*r*, θ, and Φ) based on the centroid of the tumor, and (b) City Block distance, (c) Chebyshev distance, and (d) Euclidean distance from the anterior commissure (AC) to the centroid of the tumor. The FOS features reflected the distribution of voxel values within the ROI 3D matrix and the overall information of the tumor. The shape and size features reflected the volume, surface area, and shape of the tumor. GLCM, GLRLM, GLSZM, and NGTDM were collectively referred to as texture features. The GLCM reflected the arrangement of voxels. The GLRLM reflected the arrangement of voxels with equal voxel values. The GLSZM reflected the characteristics of the homogeneous region. The NGTDM reflected the difference between each voxel in the ROI and the adjacent voxels. The wavelet features were calculated by FOS, GLCM, GLRLM, GLSZM, and NGTDM features through Coiflet 1 3D wavelet transform. The detailed information and formulas for the detection of the 734 features were published in our previous researches (19, 24).

### Feature Selection and Radiomic Signature Building

The 734 radiomic features were normalized before feature selection using the *z*-score method. A univariate analysis was used to screen the radiomic features. The criteria for screening features include (a) *p-*values of Pearson correlation coefficient <0.05 and (b) area under curve (AUC) of the radiomic features >0.6. Least absolute shrinkage and selection operator (LASSO) regression was widely used to compress the coefficients of features and select features to prevent overfitting. Logistic regression was used for data classification to build a reliable prediction model. Thereafter, LASSO and logistic regression were used to calculate the radiomic signature for epilepsy-type prediction using Glmnet package (25). Features dimension reduction and selection, i.e., univariate analyses and LASSO regression, were based on the training cohort. The optimal value of the LASSO's parameter λ was determined by leave-one-out cross-validation (LOOCV) using classification error as criterion during the training phase. We calculated the radiomic signature for the patients after determining the selected features' values using the optimal value of the LASSO's parameter λ. Radiomic signature was the linear weighting of the selected features' coefficients. Radiomic analyses of the study were implemented by MATLAB R2016a (MathWorks, Natick, MA).

### Development of an Individualized Prediction Model

Based on cohort of all patients, a multivariable logistic regression analysis was built to predict epilepsy type with clinical information, using the radiomic signature, age, gender, and tumor pathology. Akaike's information criterion was used to select the indicator with the predictive ability for building the multivariable logistic regression model (26, 27). With this radiomics-based model, we also built a novel radiomic nomogram for quantitative prediction of the epilepsy type (28).

### Performance Evaluation of the Models

The classification performance of the radiomic signature and radiomic nomogram was assessed by the receiver operating characteristic (ROC) curves and AUCs in each cohort. Calibration curves were plotted to assess the calibration of the radiomic signature and radiomic nomogram (29), accompanied by the Hosmer–Lemeshow test (30). Decision curve analyses (DCAs) determined the clinical usefulness of the radiomic signature and radiomic nomogram by quantifying the net benefits at different threshold probabilities in cohort of all patients (31).

### Statistical Analysis

Age and radiomic signature were reported as median and range. The differences between subgroups were assessed by independent samples *t*-test. Gender and histopathology were reported in frequencies and proportions, and differences between subgroups were assessed by Fisher's exact test. The statistical tests were two sided, and *p* < 0.05 were defined as significant. Nomogram building and models' validation were implemented with R software (version 3.6.1, Vienna, Austria).

## Results

### Demographic and Clinical Data

The main clinical and pathological characteristics of all 205 patients are listed in Table 1. Of the 205 enrolled patients, 139 (67.8%) had generalized and 66 (32.2%) had focal seizures. Those with generalized epilepsy accounted for 72 (70.6%) and 67 (65.0%) patients, while those with focal epilepsy accounted for 30 (29.4%) and 36 (35.0%) patients in the training and validation cohorts, respectively. There were no significant differences between the two epilepsy types based on age, gender, and tumor histopathology in cohort of all patients, training cohort, and validation cohort. However, radiomic signature was significantly different between the two epilepsy types (*p* < 0.001) and hence a potential indicator for diagnosing the types of epilepsy.

**Table 1:** Clinical characteristic of patients in the training and validation cohorts.

**Characteristics**	**All cohort** **(*****n*** **=** **205)**		***p*-value**	**Training cohort** **(*****n*** **=** **102)**		***p*-value**	**Validation cohort** **(*****n*** **=** **103)**		***p*-value**
	**G** **(*n* = 139)**	**F** **(*n* = 66)**		**G** **(*n* = 72)**	**F** **(*n* = 30)**		**G** **(*n* = 67)**	**F** **(*n* = 36)**	
Age, median (range)	37 (15–64)	39.5 (15–66)	0.181	36 (15–58)	35.5 (21–59)	0.995	41 (15–64)	44.5 (15–66)	0.118
Gender (%)			0.645			1.000			0.519
Male	85 (61)	43 (65)		44 (61)	18 (60)		41 (61)	25 (69)	
Female	54 (39)	23 (35)		28 (39)	12 (40)		26 (39)	11 (31)	
Tumor histopathology (%)			0.155			0.124			0.504
Oligodendrial glioma	97 (70)	39 (59)		48 (67)	15 (50)		49 (73)	24 (67)	
Astrocytoma	42 (30)	27 (41)		24 (33)	15 (50)		18 (27)	12 (33)	
Radiomic signature, mean ± SD	0.63 ± 1.04	−0.99 ± 1.39	<0.001	0.35 ± 0.83	−0.85 ± 0.85	<0.001	0.92 ± 1.17	−1.10 ± 1.73	<0.001

*p-values of age and radiomic signature are the results of independent-samples t-tests; p-values of gender and tumor histopathology are the results of Fisher's exact tests*.

*G, generalized; F, focal; SD, standard deviation*.

### Performance of Radiomic Signature

**Figure 2:**
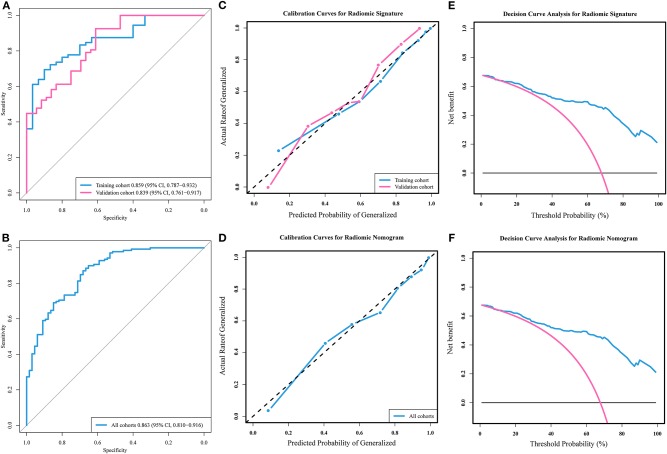
Receiver operating characteristic (ROC) curves, calibration curves, and decision curve analyses of models. **(A)** ROC curves and **(B)** calibration curves of the radiomic signature in training and validation cohorts. **(C)** Decision curve analysis of the radiomic signature. **(D)** ROC curve and **(E)** calibration curve of the radiomic nomogram in all patients' cohort. **(F)** Decision curve analysis of the radiomic nomogram.

Based on the training cohort, a logistic regression prediction model was constructed by integrating the four key radiomic features selected using the univariate analyses and LASSO regression (Table 2). The parameter λ = 0.067 was used as the optimal value. The radiomic signature for each patient in both cohorts was calculated with the train-based model. The predictive ability of the radiomic signature was interpreted from the ROC curve (Figure 2A), where it achieved a performance with classification accuracy = 80.4%, AUC = 0.859 [95% confidence interval (CI), 0.787–0.932] in the training cohort and classification accuracy = 80.6%, AUC = 0.839 (95% CI, 0.761–0.917) in the validation cohort. The radiomic signature demonstrated favorable calibration in the training and validation cohorts (Figure 2B). The *p*-values of the Hosmer–Lemeshow test for classification predictive ability of the radiomic signature were 0.12 and 0.10, respectively. The DCA showed that using radiomic signature to predict epilepsy type adds more benefit than either the treat-all-patients scheme or the treat-none scheme (Figure 2C).

**Table 2:** Four radiomic features selected by LASSO regression.

**Radiomic features**	**AUC**	***p-*values of Pearson**	**Coefficients of LASSO regression**
Coiflet_LLL_ GLSZM zone percentage	0.683	0.003	0.445876806974411
Coiflet_LLH_ NGTDM contrast	0.650	0.029	0.135681539773941
Coiflet_LHL_ GLCM maximum probability	0.685	0.023	0.336605042219162
Location features: Chebyshev distance	0.656	0.032	0.281620532274246

*p-values are the result of Pearson correlation coefficient*.

*AUC, area under curve; LASSO, least absolute shrinkage and selection operator; GLSZM, gray-level size zone matrix; NGTDM, neighborhood gray tone difference matrix; GLCM, gray-level co-occurrence matrix*.

### Performance of Radiomic Nomogram

**Figure 3:**
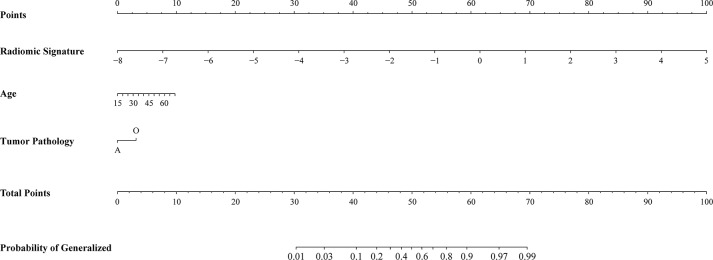
Radiomic nomogram for prediction of epilepsy type. The radiomic-based nomogram was built using radiomics signature, age, and tumor pathology data.

Radiomic nomogram for epilepsy type prediction was developed based on the radiomic signature, age, and tumor pathology data (Figure 3). It showed excellent performance in predicting epilepsy type with AUC = 0.863 (95% CI, 0.810–0.916) in cohort of all patients (Figure 2D). The calibration curve and DCA of the radiomic nomogram for the epilepsy type prediction also demonstrated favorable results (Figures 2E,F). The *p*-value of the Hosmer–Lemeshow test was 0.11.

## Discussion

This study develops and presents a quantitative and individualized epilepsy type radiomic prediction model using a series of radiomic T2-weighted imaging features associated with the type of LGG-related epilepsy. The results demonstrate that the MRI-based radiomic model could successfully stratify patients according to their epilepsy type. This easy-to-use nomogram may be a powerful clinical tool for assisting clinicians with personalized therapeutic decisions.

Treatment based on the type of LGG-related epilepsy allows for a more targeted use of antiepileptic drugs, thus minimizing therapy-related side effects in patients with LGG. However, determining the epilepsy type based on its clinical presentation imposes an apparent lag. Thus, there is a need for a clinical model capable of predicting epilepsy type before treatment initiation. In this study, a newly developed radiomic signature and radiomic nomogram predict the epilepsy type for each patient in the study. Thus, patients identified as either generalized or focal epilepsy are subjected to appropriate therapies. Therefore, the radiomic signature and radiomic nomogram provided clinicians with a reliable tool for better prediction of LGG-related epilepsy type.

For the better prediction of epilepsy type, a large number of high-throughput radiomic features that were widely used in previous radiomics studies (18) were also extracted in this study, including location features designed for brain tumor studies by Liu et al. (19). Specifically, we extracted many high-dimensional features that are intuitively challenging to be recognized by humans. Radiomic features provide abundant information on the heterogeneity and microenvironments of gliomas (32), including reliable information for its personalized treatment (33). The use of radiomics-based research in the field of oncology has indisputably impacted the survival outcomes (34, 35), lymph node metastasis (36), and treatment responses (37–39).

Furthermore, based on the quantitative MRI features, radiomic analyses have the ability to assess the clinical characteristics and molecular background of gliomas (40, 41). Therefore, this study further suggests the associations of these radiomics-based MRI features with the type of LGG-related epilepsy.

Tumor location is an influential factor associated with LGG-related epilepsy. Several MRI-based studies indicate the association between the involvement of eloquent (42), cortical (45), and insular regions (43) with epilepsy occurrence, along with the probabilistic risk atlas of LGG-related epilepsy (44). However, there is a need to investigate and predict the type of epilepsy. Furthermore, previous studies not only used the location information as categorized data but also ignored the imaging information inside the tumor area. Since various subregions of a brain lobe may differently influence the occurrence of epilepsy type, we used a quantitative description of tumor location for brain tumors. The distances from the AC to the centroid and the polar coordinates based on centroid of the tumor accurately described tumor location. These location features provide more detailed information for the radiomic prediction models in the current study.

LASSO and logistic regression are widely accepted algorithms in the field of machine learning. In this study, the 734 features extracted could cause overfitting when building the radiomic prediction model, which makes the model lose its generalization ability. Therefore, we performed feature dimension reduction and selection to detect the key features most closely related to the type of epilepsy to improve the discriminative power in the present model. The LASSO regression was used to achieve the best performance in predicting the type of LGG-related epilepsy. With features associated with epilepsy type, a prediction model was constructed using logistic regression. As a sensitive and stable machine learning method for dichotomous forecast, logistic regression has been widely used in feature-based classification. In the current study, the application of LASSO and logistic regression raises the predictive capability of the established model and consequently provided relatively high discrimination accuracies and AUCs.

There are some limitations to the present study. First, the diagnoses provided by experienced epileptologists was based on clinical presentations, and patient's epilepsy originations were unconfirmed because the stereotactic electroencephalographic data were incomplete. Second, the divergence of tumor histopathology in causing various types of epilepsy was not quantitatively assessed by the radiomic model in this study. Third, a multicenter, prospective clinical trial is required to address the limitation caused by small samples. Fourth, the interpretability of radiomic features has always been an intractable task in the study of radiomics.

## Conclusions

Radiomic location features and wavelet-based textural features are associated with the type of LGG-related epilepsy. Radiomics-based prediction models allow for non-invasive, preoperative, and low-cost prediction of epilepsy type. The results of this study suggest that radiomics could be a reliable tool for personalized treatment in patients with LGG-related epilepsy.

## Data Availability Statement

The datasets generated for this study are available on request to the corresponding author.

## Ethics Statement

The ethics committee of Beijing Tiantan Hospital approved this study, and the requirement for informed consent was waived. Our study was conducted in accordance with the Declaration of Helsinki.

## Author Contributions

YW, WW, ZL, TJ, and JT conceived of and designed the study. YW, WW, ZL, YLia, XL, YLi, ZT, and TJ collected and assembled all data. YW, WW, and ZL performed data analysis. WW, ZL, and YLia wrote the manuscript. YW revised the manuscript. All authors approved of the final manuscript.

### Conflict of Interest

The authors declare that the research was conducted in the absence of any commercial or financial relationships that could be construed as a potential conflict of interest.

## Abbreviations

AC: anterior commissure
AUC: area under curve
DCA: decision curve analysis
DICOM: Digital Imaging and Communications in Medicine
FOS: first-order statistics
GLCM: gray-level co-occurrence matrix
GLRLM: gray-level run-length matrix
GLSZM: gray-level size zone matrix
IBE: International Bureau for Epilepsy
ICC: intraclass correlation coefficient
ILAE: International League Against Epilepsy
LASSO: least absolute shrinkage and selection operator
LGG: low-grade glioma
LOOCV: leave one out cross-validation
MRI: magnetic resonance imaging
NGTDM: neighborhood gray-tone difference matrix
ROC: receiver operating characteristic
ROI: region of interest
WHO: World Health Organization
